# Raoultella ornithinolytica Urinary Tract Infection in a Patient With Triple-Negative Breast Cancer

**DOI:** 10.7759/cureus.64742

**Published:** 2024-07-17

**Authors:** Daniel T Jones, Ramaditya Srinivasmurthy, Meghana Pandit, Rachel Tovar, Liawaty Ho, Kathleen Wairimu

**Affiliations:** 1 Internal Medicine, Touro University Nevada, Henderson, USA; 2 Oncology, Comprehensive Cancer Centers, Las Vegas, USA; 3 Infectious Disease, Kathleen Wairimu, MD, Las Vegas, USA

**Keywords:** rare pathogen, case report, amoxicillin/clavulanic acid, antibiotic resistance, antibiotic susceptibility, neoadjuvant chemotherapy, immunocompromised, triple-negative breast cancer, urinary tract infection, raoultella ornithinolytica

## Abstract

Due to the challenges associated with accurately identifying *Raoultella ornithinolytica* as the causative agent in urinary tract infections (UTIs), coupled with limited guidance on treatment protocols, reports of similar cases still need to be made publicly available because of their increasing emergence. In this article, we present the first documented case of a UTI caused by *Raoultella *ornithinolytica in a patient with triple-negative breast cancer undergoing neoadjuvant chemotherapy. This case report highlights *Raoultella ornithinolytica* as an uncommon yet significant pathogen, particularly in immunocompromised patients. Given the bacterium's antibiotic resistance patterns, it emphasizes the importance of prompt, accurate identification methods and tailored treatment strategies, especially in vulnerable populations undergoing chemotherapy.

## Introduction

*Raoultella ornithinolytica*, previously classified as *Klebsiella ornithinolytica* until the genus *Raoultella* was created in 2001, is a Gram-negative, aerobic, non-motile rod-shaped bacterium within the Enterobacteriaceae family [1]. It is known for its ability to produce ornithine decarboxylase, an enzyme that converts ornithine into putrescine, a polyamine involved in various cellular functions. This bacterium also catalyzes the transformation of histidine into histamine, which can result in histamine toxicity in humans. This condition, often linked to fish consumption, is known as scombroid syndrome. *Raoultella ornithinolytica* is commonly found in various environments, including water, soil, fish, ticks, and termites, and as part of the normal flora of the gastrointestinal tract in humans and animals [2]. While it is generally not pathogenic, *Raoultella ornithinolytica* can rarely cause infections, particularly in immunocompromised individuals, those with prolonged hospital stays, catheterization, and those on mechanical ventilation [3]. In a review of 112 patients with *Raoultella ornithinolytica* infections, the most prevalent type of infection was urinary tract infection (UTI), observed in 36 cases (32%). This was followed by respiratory infections in 27 cases (24%), gastrointestinal infections in 16 cases (14%), and skin/wound infections in 15 cases (13%) [4].

*Raoultella ornithinolytica* poses identification challenges through conventional biochemical and phenotypic tests and is thus underreported and frequently misidentified as *Klebsiella* spp. due to their genetic and phenotypic parallels. This emphasizes the importance of accurate diagnostic methods to distinguish between these pathogens for effective treatment [5]. *Raoultella ornithinolytica* is identifiable through a combination of biochemical tests, morphological observation, and molecular techniques such as 16S rRNA sequencing [6]. Research also highlights the antibiotic resistance patterns of *Raoultella ornithinolytica*, showing resistance to multiple antibiotics, including beta-lactams, which further complicates treatment protocols [7]. The general consensus advises treating a *Raoultella ornithinolytica* UTI with antibiotics such as amoxicillin combined with clavulanic acid for a duration of 10-14 days [8]. In cases of emerging multi-drug-resistant strains, antibiotics such as ceftazidime-avibactam are used as second-line agents for broad-spectrum treatment [9].

This case report is of a patient who developed a UTI due to *Raoultella ornithinolytica* during the course of her neoadjuvant chemotherapy regimen. The objective is to highlight the challenges in accurately identifying this pathogen and its diverse virulent factors, discuss its antibiotic resistance patterns, and emphasize the importance of tailored treatment strategies for immunocompromised patients.

## Case presentation

An 81-year-old woman with a past medical history of stage 1 ER-negative, PR-positive, HER2-negative left breast cancer (2014), right partial nephrectomy for a stage I clear cell carcinoma of the kidney (2015), and a recent diagnosis of triple-negative contralateral breast cancer (2024) presents for evaluation. The patient is also positive for a PAI-1 mutation and is a carrier of a MUTYH mutation. The patient underwent a bilateral diagnostic mammogram and breast ultrasound, revealing a new dense mass measuring 1.7 cm × 1.5 cm in the right upper outer quadrant. A biopsy was recommended and subsequently performed. Pathology results indicated the presence of grade 2 of 3 invasive ductal carcinomas: ER-negative, PR-negative, and HER2-negative (0% = triple-negative breast cancer). She denied experiencing chest pain, dyspnea, abdominal pain, bone pain, or headaches. She underwent a PET/CT scan, which identified a nodule in the right breast with no evidence of distant metastases. A breast MRI revealed a 2.3 cm mass in the right breast with no lymphadenopathy in the right axilla. She received neoadjuvant chemotherapy consisting of carboplatin, weekly paclitaxel, and pembrolizumab the previous week, which she tolerated well. The patient denied experiencing nausea and vomiting but reported mild constipation, feelings of dehydration, and mild headaches, which resolved with acetaminophen. She received her third dose of carboplatin, paclitaxel, and pembrolizumab and has thus far tolerated the chemotherapy regimen, albeit complicated by mild fatigue. She received her fourth dose and complained of fatigue and an increased urinary frequency. A urinalysis revealed a positive urine culture of *Raoultella ornithinolytica*. The patient admits to consuming a wide range of fish three to five times a week in portions of 6-8 oz. The urine culture results indicated a colony count of greater than 100,000 colony-forming units per milliliter. The patient was prescribed amoxicillin-clavulanate oral tablets, each containing 875 mg of amoxicillin and 125 mg of clavulanate, and instructed to take one tablet orally every 12 hours for a duration of 10 days. The patient's symptoms had resolved by the time of the follow-up visit.

## Discussion

*Raoultella ornithinolytica* represents an exceptionally rare pathogen, with a limited number of documented human infections reported in the medical literature. Both our case and the cases reported by Hadano et al. involve immunocompromised patients with cancer. *Raoultella ornithinolytica*, in all cases, showed resistance to ampicillin and susceptibility to amoxicillin/clavulanate, cefepime, and gentamicin (Table 1). All cases demonstrated positive clinical responses to tailored antibiotic therapy [10]. *Raoultella ornithinolytica* is commonly found in various environments, including water, soil, fish, ticks, and termites, and as part of the normal flora of the gastrointestinal tract in humans and animals [2]. This case involves an immunocompromised patient who consumes various types of fish three to five times a week, typically in portions of around 6 to 8 oz, and who developed a *Raoultella ornithinolytica* UTI. This case underscores the need for further research into the potential role of fish consumption as a risk factor for UTIs caused by *Raoultella ornithinolytica*, especially since there are no documented cases of such infections in immunocompromised patients with a diet including fish. Typically, when encountered, *Raoultella ornithinolytica* primarily manifests in healthcare-associated infections, particularly among immunocompromised and hospitalized patients. In the study by Hadano et al., three patients were documented with *Raoultella ornithinolytica* bacteremia, each associated with different types of biliary tract infections. The first patient experienced cholecystitis, the second had cholangitis, and the third developed an intra-abdominal abscess [10].

**Table 1:** Antibiotic susceptibility pattern of Raoultella ornithinolytica using the disc diffusion test

Antibiotic	Sensitivity
Amoxicillin/clavulanic acid	Susceptible
Ampicillin	Resistant
Cefepime	Susceptible
Cefuroxime	Susceptible
Gentamicin	Susceptible
Nitrofurantoin	Susceptible
Tetracycline	Resistant
Tobramycin	Susceptible

This Gram-negative bacterium showcases a diverse array of virulence factors, encompassing adhesins, toxins, and resistance mechanisms, which bolster its pathogenicity. Notably, its capacity to form biofilms on medical devices poses significant challenges in treatment and eradication, resulting in persistent infections and escalated healthcare expenditures [11]. *Raoultella ornithinolytica*’s pathogenicity and antibiotic resistance mechanisms assume importance in devising effective strategies for the prevention, diagnosis, and management of its infections [3]. Despite its rarity, heightened vigilance and ongoing research efforts are essential to comprehensively understanding the epidemiology and clinical implications associated with *Raoultella ornithinolytica* infections.

Diagnosing *Raoultella ornithinolytica* infections involves isolating the bacterium from clinical specimens such as blood, urine, or respiratory secretions through standard microbiological techniques, including culture and biochemical identification. Due to its similarities to other *Enterobacteriaceae* spp., misidentification can occur, leading to diagnostic errors. *Raoultella ornithinolytica* is frequently misidentified as a *Klebsiella* spp., particularly *Klebsiella pneumoniae*. This misidentification occurs because of their close genetic and phenotypic similarities, making it challenging to distinguish between them using standard microbiological techniques. Both bacteria exhibit similar morphological characteristics in laboratory cultures and share biochemical properties, such as lactose fermentation, leading to comparable outcomes in biochemical tests. Conventional microbiological techniques often lack the specificity to distinguish between these species, and many clinical laboratories use automated systems that default to identifying *Raoultella* spp. as *Klebsiella pneumonia* [6]. Utilizing advanced molecular methods such as polymerase chain reaction assays targeting specific *Raoultella ornithinolytica* genetic markers can enhance accuracy and expedite diagnosis. Additionally, matrix-assisted laser desorption/ionization time-of-flight mass spectrometry (MALDI-TOF MS) has emerged as a valuable tool for rapid and reliable species identification in clinical microbiology laboratories [12]. MALDI-TOF MS would generate a unique protein mass spectrum profile for the bacterial sample. This profile would be compared against a reference database of known bacterial spectra [13].

The treatment of *Raoultella ornithinolytica* infections involves antibiotic therapy tailored to the susceptibility profile of the isolated strain. The first-line treatment for a *Raoultella ornithinolytica* infection is antibiotics such as amoxicillin combined with clavulanic acid for a duration of 10-14 days [8]. Other first-line agents include broad-spectrum antibiotics such as carbapenems or third-generation cephalosporins, which have demonstrated efficacy against Enterobacteriaceae. Strains of *Raoultella ornithinolytica* generally exhibit susceptibility to amoxicillin with clavulanic acid, piperacillin, piperacillin-tazobactam, second- to fourth-generation cephalosporins, carbapenems, aminoglycosides, fluoroquinolones, trimethoprim/sulfamethoxazole, and tigecycline [1]. With the emergence of multidrug-resistant strains, including those producing extended-spectrum β-lactamases or carbapenemases, alternative options may be necessary. In such cases, newer antibiotics like ceftazidime-avibactam, meropenem-vaborbactam, or cefiderocol can be considered second-line agents, offering activity against resistant pathogens. Additionally, the use of combination therapy or adjunctive agents such as colistin may be warranted in severe or complicated infections [12]. Clinicians should carefully evaluate the patient's clinical condition, the severity of infection, and local antimicrobial resistance patterns when selecting appropriate treatment regimens.

*Raoultella ornithinolytica* has demonstrated a concerning trend of antimicrobial resistance, posing challenges in clinical management. Case reports have highlighted its ability to develop resistance mechanisms against multiple antibiotic classes, including β-lactams, fluoroquinolones, and aminoglycosides [14]. These cases underscore the clinical significance of *Raoultella ornithinolytica* as a multidrug-resistant pathogen and emphasize the importance of antibiotic stewardship and ongoing surveillance to combat the emergence and spread of resistance in healthcare settings.

## Conclusions

This case report sheds light on the intricacies of diagnosing and managing UTIs caused by *Raoultella ornithinolytica*, particularly in immunocompromised patients undergoing chemotherapy. The rarity of such infections, coupled with challenges in accurate identification and limited treatment guidelines, underscores the significance of this case. By presenting the first documented instance of a UTI due to *Raoultella ornithinolytica* in a patient with triple-negative breast cancer on neoadjuvant chemotherapy, this report highlights the importance of vigilance in identifying uncommon pathogens, especially in vulnerable patient populations. The successful resolution of symptoms following tailored antibiotic therapy further emphasizes the importance of individualized treatment strategies guided by antibiotic susceptibility testing. This case contributes to the existing medical literature by enhancing our understanding of Raoultella ornithinolytica infections and emphasizing the need for continued research to optimize diagnosis and management approaches for such cases.
